# Barrier inhomogeneities limited current and 1/*f* noise transport in GaN based nanoscale Schottky barrier diodes

**DOI:** 10.1038/srep27553

**Published:** 2016-06-10

**Authors:** Ashutosh Kumar, M. Heilmann, Michael Latzel, Raman Kapoor, Intu Sharma, M. Göbelt, Silke H. Christiansen, Vikram Kumar, Rajendra Singh

**Affiliations:** 1 Department of Physics, Indian Institute of Technology Delhi, New Delhi-110016, India; 2 Nanoscale Research Facility, Indian Institute of Technology Delhi, New Delhi-110016, India; 3 Max Planck Institute for the Science of Light, Günther-Scharowsky-Straße 1/Bau 24, 91058 Erlangen, Germany; 4 Friedrich-Alexander-Universität Erlangen-Nürnberg (FAU), Institute of Optics, Information and Photonics, Staudtstr. 7/B2, 91058 Erlangen, Germany; 5 Institute of Nano-Architectures for Energy Conversion, Helmholtz - Zentrum Berlin für Materialien und Energie GmbH, Hahn-Meitner-Platz 1, 14109 Berlin, Germany

## Abstract

The electrical behaviour of Schottky barrier diodes realized on vertically standing individual GaN nanorods and array of nanorods is investigated. The Schottky diodes on individual nanorod show highest barrier height in comparison with large area diodes on nanorods array and epitaxial film which is in contrast with previously published work. The discrepancy between the electrical behaviour of nanoscale Schottky diodes and large area diodes is explained using cathodoluminescence measurements, surface potential analysis using Kelvin probe force microscopy and 1ow frequency noise measurements. The noise measurements on large area diodes on nanorods array and epitaxial film suggest the presence of barrier inhomogeneities at the metal/semiconductor interface which deviate the noise spectra from Lorentzian to 1/*f* type. These barrier inhomogeneities in large area diodes resulted in reduced barrier height whereas due to the limited role of barrier inhomogeneities in individual nanorod based Schottky diode, a higher barrier height is obtained.

GaN nanostructures are intensively investigated due to advantages like capabilities of incorporation of both n- and p-type dopants^1^,^2^, strain relaxation when grown on lattice mismatched substrates^3^,^4^, dislocation free structures^5^ and enhanced light absorption^6^. Accordingly, GaN nanostructures have been functionalized in various nanoscale devices such as light emitting diodes (LEDs)^7^,^8^, p-n junctions^9^,^10^, photodetectors^11^,^12^, field-effect transistors^13^,^14^, lasers^15^,^16^ and solar cells^17^,^18^. To realize a nanoscale device based on GaN nanorods (NRs), a thorough understanding of the current transport across interface is required as models which are used for explaining electrical transport in epitaxial films are not fully applicable to nano-devices because of small dimensions^19^,^20^. In existing literature, metals like Al, Ti/Pt, Pt etc. have been used as Schottky contacts on GaN nanostructures grown using bottom-up approach^21^,^22^,^23^,^24^,^25^. In these reports, ideality factor (*η*) and Schottky barrier height are reported to vary between 6–18 and 0.2–0.6 eV, respectively. From technological point of view, values of *η* and barrier height need to be significantly improved if one wants to fabricate a good quality Schottky barrier diode of nanoscale dimensions.

In nanostructures with diameter comparable to carrier diffusion length and higher surface-to-volume ratio, surface states play a crucial role in deciding electronic transport. Thus, electronic properties of nanostructures depend strongly on surface effects and hence on native surface states^26^. Contrast to larger area diodes, effect of barrier inhomogeneities and interface states on electrical behaviour on nanoscale diodes is expected to be different due to reduced dimensions. Therefore, for employing semiconductor nanostructures into promising device technology, the correlation between surface properties, barrier inhomogeneities, interface states and electrical transport properties needs to be understood i.e. how the surface of individual nanostructure is contributing to the device characteristics.

Present work compares the electrical behaviour of nanoscale Schottky diodes formed on individual GaN nanorod with large area diodes formed on array of GaN NRs and GaN epitaxial film. Comprehensive characterizations using micro-Raman spectroscopic, micro-photoluminescence (μ-PL), cathodoluminescence (CL) measurements and Kelvin probe force microscopy (KPFM) are performed to examine and compare the optical and surface potential properties of a single GaN nanorod as well as NRs array with respect to parent epitaxial film. Due to scattering from surface states as well as large surface-to-volume ratio, low frequency noise fluctuations are expected to be pronounced in nanoscale devices in comparison to planar devices. Thus, 1/*f* noise measurements are also performed on diodes fabricated on epitaxial film as well as on array of NRs.

## Results and Discussion

### Morphological and optical studies of GaN NRs

**Figure 1:**
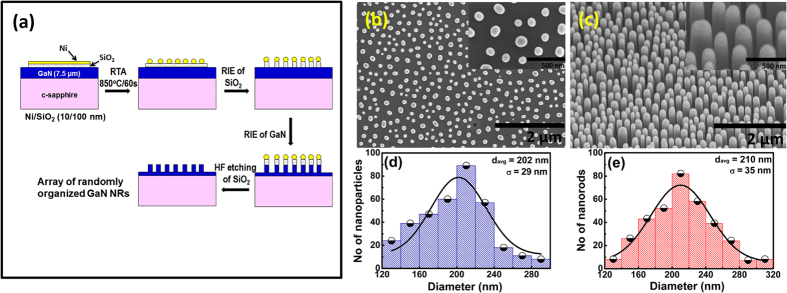
(**a**) Schematic of GaN NRs fabrication process using Ni nanomasking and reactive ion etching: a top-down approach. (**b**) FESEM image of Ni NPs formed after rapid thermal annealing Ni film at 850 °C for 1 min. (**c**) FESEM image of vertically standing GaN NRs after selective RIE of SiO_2_ and GaN. Size distributions of NPs and NRs are shown in (**d**,**e**), respectively. The NRs are about 1.2 μm in length and 210 nm (standard deviation of 35 nm) in diameter. Insets of (**b**,**c**) show magnified view of NPs and NRs, respectively.

The Schematic of the GaN NRs fabrication process using Ni nanomasking and reactive ion etching is shown in Fig. 1(a). The field emission scanning electron microscopy (FESEM) images of Ni nanoparticles (NPs) and GaN NRs are shown in Fig. 1(b,c), respectively, with inset showing the magnified view. Size distributions of NPs and NRs are shown in Fig. 1(d,e) where average diameters (d_avg_) of NPs and NRs are found to be 202 nm (with a standard deviation of 29 nm) and 210 nm (with a standard deviation of 35 nm), respectively. Almost similar diameters of NPs and NRs confirmed that NPs acted as masks during inductively coupled plasma-reactive ion etching (ICP-RIE) of SiO_2_ and GaN. Density of NRs is calculated to be ~1.86 × 10^9^ *cm*^−2^.

To investigate strain relaxation in GaN NRs, Raman measurements have been performed in 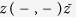 backscattering geometry (z axis is parallel to c-axis) on GaN epitaxial film and GaN NRs. The compressive stress is relaxed by 0.90 GPa in NRs as compared to epilayer. Raman measurements and stress calculation are given in Supplementary information. The stress relaxation calculated from Raman measurements is found to be in agreement with results of photoluminescence (PL) measurements. Also, PL intensity of near band-edge (NBE) luminescence is enhanced in the case of NRs which suggests that these NR arrays may have potential applications in GaN based nanoscale optoelectronic devices. Results and discussion of PL measurements are given as Supplementary information.

**Figure 2:**
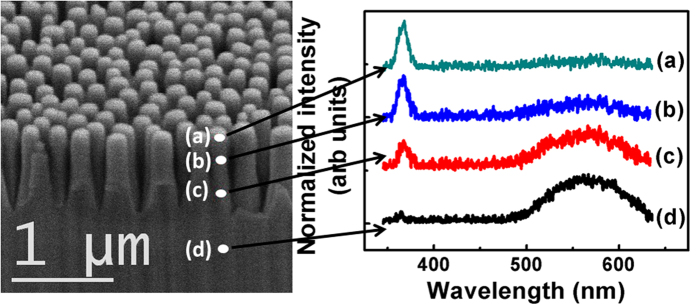
FESEM image of vertically standing GaN NRs (60° tilted view) and corresponding CL spectra of a single GaN nanorod taken at four positions: (**a**) top, (**b**) centre and (**c**) bottom of the nanorod, and (**d**) underlying GaN epitaxial layer. Relatively less intensity of YL at the top of nanorod indicates that top is relatively defect free as compared to centre, bottom and underlying layer. This is also expected as top of the rod is not exposed to ICP-RIE as it is covered by SiO_2_ and Ni NPs.

In present work, GaN NRs are fabricated using ICP-RIE process which can possibly lead to surface modifications of NRs. Therefore, distribution of defects along a single GaN nanorod is studied by CL measurements at four different positions: top, centre and bottom of the rod and also at underlying GaN epitaxial film as shown in Fig. 2. The CL spectra have same features as PL as NBE luminescence in all cases is close to 365 nm (3.4 eV). The other feature of CL spectra is a broad peak centred at 565 nm (2.2 eV) commonly termed as yellow luminescence (YL), which has been related to the defects^27^,^28^,^29^,^30^,^31^. These defect states are formed on the surface of the GaN nanorod due to Ga or N termination, surface reconstructions, relaxation, dangling bonds or damages introduced during RIE. When electron beam is focussed at the top of the nanorod, CL spectrum is dominated by the NBE luminescence while YL is almost negligible. As electron beam is moved to the centre and then to the bottom of the nanorod, YL started to increase while intensity of near band edge luminescence (as compared to YL intensity) almost remained the same. Also, YL is seen to dominate the CL spectrum of the underlying GaN epitaxial layer. This suggests that there is a clear transition from NBE centred at 364 nm to YL centred around 565 nm as a function of position (from top of GaN nanorod to underlying GaN epilayer). It is evident from the intensity of YL that top of the GaN nanorod is relatively less defective as compared to centre and bottom of the rod as well as epilayer. This is expected as top of the NRs is protected by Ni NPs and SiO_2_ layer during ICP-RIE process.

### KPFM studies of GaN epitaxial film and an individual nanorod

**Figure 3:**
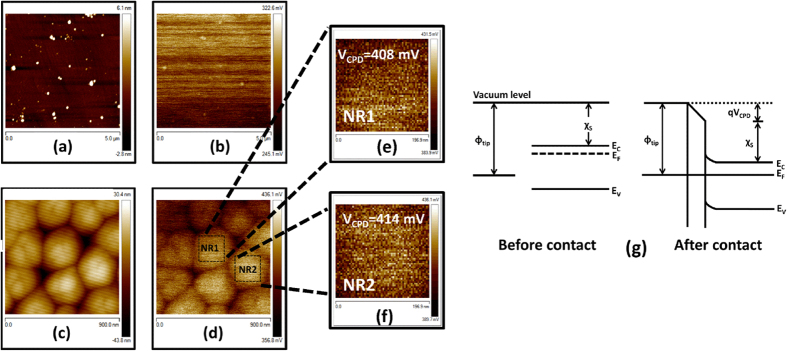
(**a**) AFM topographical image while (**b**) shows corresponding *V*_*CPD*_ image for GaN epitaxial film. RMS roughness is obtained as 0.9 nm which indicated good surface quality. Mean *V*_*CPD*_ obtained from nanoanalysis software is found to be 285 mV. Topographical and corresponding *V*_*CPD*_ images for NRs are shown in (**c**,**d**), respectively. From (**d**), Square portions (NR1 and NR2) of dimensions 197 × 197 nm^2^ are selected and respective *V*_*CPD*_ images are shown in (**e**,**f**). The values of *V*_*CPD*_ for NR1 and NR2 are found to be 408 and 414 mV, respectively. Band alignment of metallic tip-semiconductor (n-type) system before and after contact is shown in (**g**). As tip and semiconductor comes in contact, electrons flow from n-type semiconductor to tip giving rise to a contact potential difference, *V*_*CPD*_.

While comparing electrical transport at nanoscale with the bulk, the variation in surface properties should be considered. As NRs in the present work are fabricated using dry ICP-RIE followed by wet etching in HF, their surface properties are expected to be different in comparison to as-grown GaN epitaxial film. Any small modification in surface can alter surface potential which in turn changes the Fermi level position in the semiconductor^32^,^33^,^34^. We have performed surface potential measurements on GaN epitaxial film and NRs using KPFM technique. KPFM measures contact potential difference (*V*_*CPD*_) which is given as *V*_*CPD*_ = (*ϕ*_*tip*_ − *ϕ*_*sample*_)/*q* where *ϕ*_*tip*_ and *ϕ*_*sample*_ are work functions of conductive tip and sample^32^,^35^. Prior to KPFM measurements on GaN epilayer and NRs, value of *ϕ*_*tip*_ is obtained by calibration with respect to freshly cleaved highly oriented pyrolytic graphite (HOPG) used as a reference material. The value of *ϕ*_*sample*_ for HOPG is taken as 4.60 eV^36^,^37^. The value of *V*_*CPD*_ between tip and HOPG is found to be 0.05 eV, which gives *ϕ*_*tip*_ = 4.65 *eV* for present study. Same tip is used for KPFM measurements on as-grown epitaxial film as well as NRs. For GaN epitaxial films, 5 × 5 μm^2^ scans across 3 different regions are obtained. In order to determine an absolute value of surface potential, the mean of *V*_*CPD*_ across all points is calculated by the Nanoscope Analysis software. Fig. 3(a) shows one typical AFM image of GaN epitaxial film with root mean square (RMS) roughness of 0.9 nm while Fig. 3(b) shows corresponding *V*_*CPD*_ image where mean *V*_*CPD*_ is found to be 285 mV. For other two regions, mean *V*_*CPD*_ values are found to be 301 mV each. Almost similar values of *V*_*CPD*_ (285, 301 and 301 mV) indicate high surface uniformity of GaN epitaxial film. For vertical GaN NRs, 900 × 900 nm^2^ scans are obtained across 3 different locations. Fig. 3(c) represents one typical AFM image of GaN NRs with RMS roughness of 9.9 nm while Fig. 3(d) shows corresponding *V*_*CPD*_ image with mean *V*_*CPD*_ equal to 398 mV. For GaN NRs, mean *V*_*CPD*_ value of complete sample is likely to be different from *V*_*CPD*_ of single nanorod due to contributions from the air gap between two individual NRs. Therefore, to obtain *V*_*CPD*_ across a single nanorod, square sections of area 197 × 197 nm^2^ labeled as NR1 and NR2 (see Fig. 3(d)) are selected on individual nanorod using nano analysis software. The mean *V*_*CPD*_ values are found to be 408 mV (for NR1) and 414 mV (for NR2) as shown in Fig. 3(e,f). The scans of area 900 × 900 nm^2^ are also obtained across two more randomly selected regions. Across each region, two NRs are selected and mean *V*_*CPD*_ value across each individual nanorod is evaluated in the same manner as discussed above. The mean *V*_*CPD*_ values across these four individual NRs are calculated to be 411, 389, 438 and 448 mV. Hence, the mean *V*_*CPD*_ values across individual NRs are found to higher as compared to *V*_*CPD*_ across as-grown epitaxial films.

KPFM technique works on the electrostatic interaction between the tip and sample due to difference in their Fermi energy levels. When tip and sample are electrically isolated, their vacuum levels match but Fermi energy levels are at different positions. On contacting, charge transfer takes place to achieve equilibrium (Fermi level at same position) causing misalignment in vacuum energy levels on both sides. This causes the formation of contact potential difference. Energy band diagram of tip and sample (n-type semiconductor in present study) before and after contact is shown in Fig. 3(g). Under equilibrium^32^,^33^


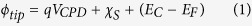


In above equation, the semiconductor surface band bending is not considered. Hence *V*_*CPD*_ images obtained from KPFM can be used to obtain doping concentration profile as^32^,^33^


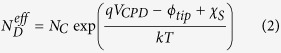


where 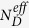 is the effective donor concentration and *N*_*C*_ the effective density of states of GaN conduction band and is equal to 2.6 × 10^18^ cm^−3 ^38. From above relation, it is clear that higher the *qV*_*CPD*_, higher the effective donor concentration. For GaN epitaxial film, *V*_*CPD*_ is 296 mV (mean of 285, 301 and 301 mV), corresponding 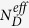 is found to be 7 × 10^15^ *cm*^−3^ as calculated from Eq. 2. The effective doping concentration of GaN epitaxial film calculated by KPFM measurements is comparable with the doping concentration measured from Hall measurements (2 × 10^16^ *cm*^−3^). From mean *V*_*CPD*_ values of individual NRs, 408 mV (for NR1) and 414 mV (for NR2) as shown in Fig. 3(e,f), corresponding 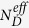 are calculated to be 5 × 10^17^ *cm*^−3^ (for NR1) and 7 × 10^17^ (for NR2). For other individual NRs, 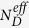 are found to similar. Hence, 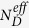 across a single GaN nanorod is about two orders higher in magnitude as compared to GaN epilayer. This can significantly affect the electrical properties of nanoscale devices. Various authors attributed increase in doping concentration to ICP-RIE processing^39^,^40^,^41^. Chen *et al*.^39^ studied effect of RIE in GaN epitaxial layers and found that ion bombardment during RIE process may increase N vacancies that can enhance doping concentration. Jang *et al*.^40^ investigated effect of Cl_2_ based ICP-RIE on n-type GaN films and found an increment in the binding energy of Ga-O bond and Ga/N atomic ratio after ICP plasma treatment. This results in creation of N vacancies which acted as donors for electron at GaN surface causing an increase in effective electron concentration. Increase in electron concentration after SiCl_4_ RIE treatment is also reported by Selvanthan *et al*.^41^ where blue shift of Ga-N (Ga3d) peak towards higher binding energy shifts the Fermi level towards conduction band. This Fermi level shift is related to increased electron concentration due to creation of N vacancies. Hence, we attribute increased 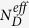 in NRs as compared to epitaxial film to N vacancies created during ICP-RIE processing.

It is well known that surface states at semiconductor surface induces band bending (band bends upward for n-type semiconductor and downwards for p-type semiconductor) which affect the electrical behaviour of semiconductor devices^42^,^43^. From KPFM results, the surface potential of NRs is higher than un-etched an epitaxial film which indicates different surface state density is NRs in comparison to epitaxial films. Therefore, surface band bindings are expected to be different in both the cases and should be calculated for better understanding of bulk and nanoscale electronic transport. On considering surface band bending, Eq. 1 modifies as 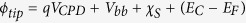, where *V*_*bb*_ is surface band bending. On using calculated values of *V*_*CPD*_ and (*E*_*C*_ − *E*_*F*_), the *V*_*bb*_ values are calculated to be 1.304 and 1.062 eV for GaN epilayer and GaN NRs, respectively. The surface band bending (*V*_*bb*_) is related to carrier concentration (*N*_*D*_) and surface state density (*N*_*ss*_) as 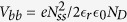, where other symbols have usual meanings^42^. The *N*_*SS*_ is calculated to be 1.24 × 10^10^ cm^−2^ and 1.1 × 10^11^ cm^−2^ for GaN epitaxial film and GaN NRs, respectively. As GaN NRs are fabricated using ICP-RIE process, higher *N*_*SS*_ in NRs is expected due to damages caused during ICP-RIE. Due to higher *N*_*SS*_ in NRs, the electrical behaviour of nanoscale devices will be different in comparison to epitaxial devices. To confirm this, the electrical behaviour of Schottky diodes on individual NR, array of NRs and epitaxial films is investigated and will be discussed in the following sections of the manuscript.

### Electrical and 1/*f* noise characteristics

#### Electrical properties of Schottky diode on a single vertically standing GaN nanorod

**Figure 4:**
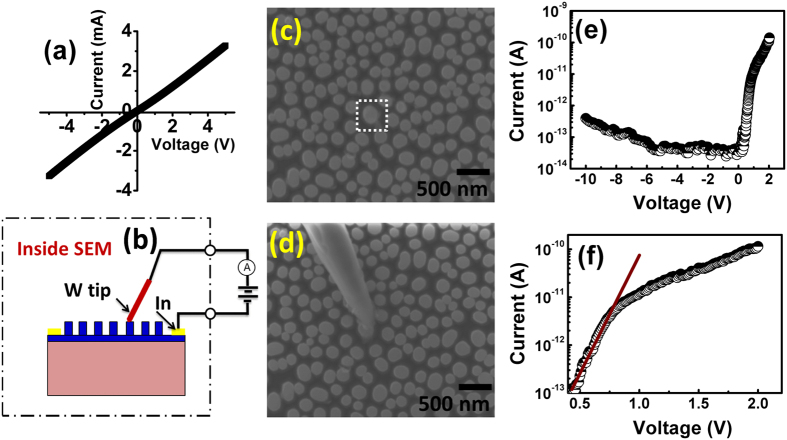
(**a**) Current-voltage (I-V) characteristics of In/GaN Ohmic contacts using two-probe method. Schematic of electrical characterization of nanoscale W/GaN Schottky contact is shown in (**b**). Top view FESEM images of vertically standing NRs before and after contact with W tip inside FESEM are shown in (**c**,**d**), respectively. Rectangular box in (**a**) shows the nanorod selected for electrical contact. I-V characteristics of nanoscale W/GaN Schottky diode on a single vertically standing GaN nanorod are shown in (**e**) whereas (**f**) shows initial region on expanded scale fitted in accordance with thermionic emission model.

For device fabrication, Ohmic contacts are made with Indium using soldering method on rectangular portions where NRs are not fabricated as shown in Fig. 1(a). The Ohmic behaviour of Indium contacts on GaN is verified by I-V measurement in two-probe configuration. The linear behaviour of I-V plot as shown in Fig. 4(a), confirms that indium contacts served as Ohmic contacts on GaN epitaxial film. Schottky contact on a single GaN nanorod is formed by contacting a single nanorod with Tungsten (W) tip of 100 nm radius of curvature using a nano-prober assembly (Kammrath & Weiss) inside SEM (FEI Strata DB235). Due to the higher work function of W^44^,^45^ in comparison to electron affinity of GaN, a Schottky contact is formed between W tip and GaN nanorod. Schematic of electrical characterization of nanoscale Schottky diode is shown in Fig. 4(b). The top-view of the nanorod before the contact is shown in Fig. 4(c) while Fig. 4(d) shows the same area after the tip is brought in contact with one nanorod. I-V characteristic of a nano-Schottky diode based on a single GaN nanorod at room temperature on semi-log scale is shown in Fig. 4(e). Forward I-V characteristic is re-plotted in Fig. 4(f) on expanded scale. As it is clear from Fig. 4(f), a fast rise in the current is followed by series resistance limited current.

According to thermionic emission model, current flowing across a metal-semiconductor interface is given as^46^


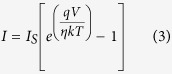



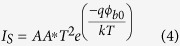


where *η* is ideality factor, *ϕ*_*b*0_ the zero bias Schottky barrier height, A the area of contact, V the applied voltage, T the measurement temperature in Kelvin, k the Boltzmann constant. A* is Richardson’s constant ~ 26.8 Acm^−2^K^−2^ for GaN^47^. From slope and intercept of Fig. 4(f), η and *ϕ*_*b*0_ for nano-Schottky barrier diode on single GaN nanorod are found to be 3.3 and 0.68 eV, respectively at room temperature. The radius of curvature of the W tip is 100 nm, but exact area of the tip contacting the nanorod is not known. While calculating *ϕ*_*b*0_, area of W tip in contact with GaN nanorod is assumed to be π(50 nm)^2^. This is acceptable as an error of even 50 nm in radius of contact area with nanorod would lead to a change in *ϕ*_*b*0_ by 0.03 eV only. As work function of W (*ϕ*_*m*_) lies between 4.7–4.9 eV^44^,^45^ and electron affinity (*χ*_*s*_) of GaN is close to 4.2 eV^48^ therefore Schottky barrier height should lie between 0.5–0.7 eV as predicted by Schottky-Mott model (*ϕ*_*B*_ = *ϕ*_*m*_ − *χ*_*s*_). The barrier height in present study is in agreement with Schottky-Mott model. It is noteworthy here that, our values of η and *ϕ*_*b*0_ are much improved as compared to earlier reports of nano-Schottky diode on GaN NWs and NRs^21^,^22^,^23^,^24^,^25^.

Ideality factor of 3.3 in nano-Schottky diode on a single GaN nanorod is larger than unity which suggests that thermionic emission is not the dominant mechanism at nanoscale interface and role of tunneling, electrical dipole formation, interface states, barrier inhomogeneities etc. have to be considered to explain this non-ideal behaviour^46^. It is already demonstrated that properties of diodes on nanoscale dimensions deviate significantly as compared to diodes formed on bulk or epitaxial films because of the fact that tunneling current becomes significant when diode size is less than or comparable with zero bias depletion width (*δ*)^20^. Zero bias depletion width (*δ*) can be calculated as^20^


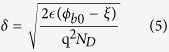


here, *ϕ*_*b*0_ is the Schottky barrier height at zero bias, *ξ* = (kTln(*N*_*C*_/*N*_*D*_)) is position of the conduction band minimum with respect to Fermi level position (E_F_) in GaN nanorod, where *N*_*C*_ is equal to 2.6 × 10^18^ cm^−3^ 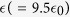 is the permittivity of GaN^38^. *N*_*D*_ is doping concentration in a GaN nanorod and is equal to 5 × 10^17^ cm^−3^ as measured by KPFM measurement across a single NR. Using Eq. 5 and *ϕ*_*b*0_ = 0.68 eV, *δ* is calculated to be ~36 nm. As radius of curvature of the contact between W tip and GaN, which is assumed to be 50 nm, is comparable with *δ*, therefore tunneling current could become significant at nanoscale W/GaN metal-semiconductor interface deviating ideality factor from unity.

From KPFM measurements, effective doping concentration across a single NR is about two orders higher in magnitude as compared to epilayer, this reduce the width of depletion layer. The reduction in depletion width increases the probability of tunneling of carriers across the barrier which results in increase in ideality factor. Also, in present study, a Schottky contact of nanoscale dimensions is formed by contacting a single rod with very sharp tip, this induces a high electric field on the top of GaN nanorod beneath the contact due to sharp curvature. This results into enhanced tunneling which causes ideality factor to be larger as compared to W/GaN epitaxial film diodes. In addition, trap-assisted tunneling may also cause ideality factor to deviate from unity, but in present study, *δ* is smaller as compared to height of NRs which is about 1.2 μm. As electrical contact is made on the top which is quite far from bottom of the nanorod and GaN underlying GaN where defects are localized (as revealed by CL measurement), therefore, role of trap-assisted tunnelling in deciding current transport across metal-semiconductor interface (at top of the GaN nanorod) is expected to be limited and can be neglected. Another cause for ideality factor deviating from unity is the formation of electric dipole layer at W/NR interface. Schottky contact is formed with W tip on top of the nanorod where Ga or N terminated atoms are always present. As electronegativity of N atoms in contact with W atoms is higher than Ga atoms, an interfacial dipole layer is formed. This dipole layer is associated with dipole moment and electric field directed towards GaN nanorod and W tip, respectively. This electric field results in effective reduction of barrier height which causes ideality factor to deviate from unity. The effective reduction of barrier height due to electric dipole formation is also reported earlier in Au/GaAs NWs^49^.

#### Electrical properties of Schottky barrier diodes on GaN NRs array and GaN epitaxial film

**Figure 5:**
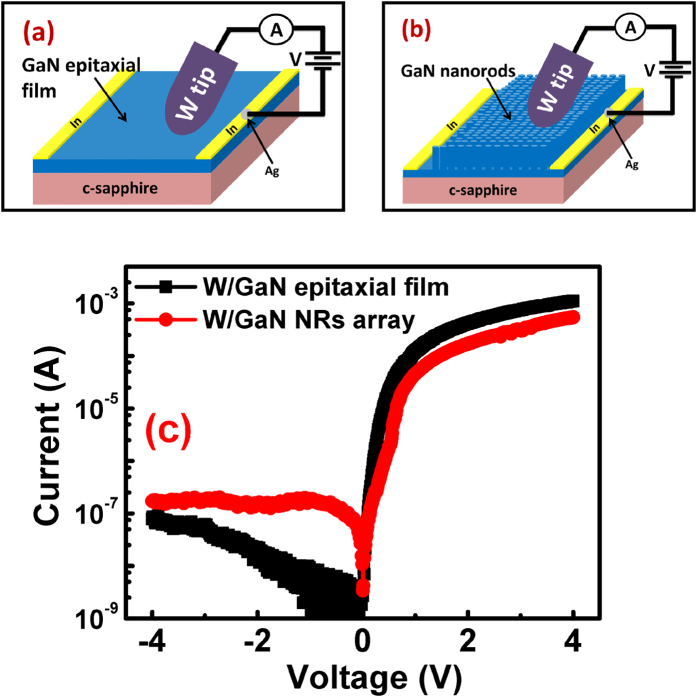
(**a**,**b**) Schematics of electrical characterization of W/GaN epitaxial film and W/GaN NRs diodes, respectively. I-V characteristics for W/GaN epitaxial film diode and W/GaN NRs array diodes are presented in (**c**).

To compare electronic transport at nanoscale with the bulk, Schottky diodes are also formed on bare GaN epilayer and GaN NRs array as shown schematically in Fig. 5(a,b), respectively. A W tip of radius of curvature 40 μm is used for contacting the sample in both the cases. Indium contacts deposited on sides of the sample served as Ohmic contacts. It is important to note that W tip and Indium contacts are selected so that one can compare the electric transport on W/GaN epitaxial film (or W/GaN NRs) diode with W/GaN nanoscale diode realized on a single GaN nanorod. I-V characteristics are taken by contacting W tip at four different positions on GaN epitaxial film as on GaN NRs array. I-V characteristics for one of such positions for W/GaN epilayer diode and W/GaN NRs diode are shown in Fig. 5(c).

The values of η and *ϕ*_*b*0_ in each case are calculated using Eqs 3, 4. The area of W tip in contact with GaN epitaxial film (or GaN NRs) is not exactly known; hence it is taken approximately as π(20 μm)^2^ for calculating *ϕ*_*b*0_. It is reasonable as even fifty percent error in π(20 μm)^2^ can change *ϕ*_*b*0_ by 0.03 eV only. For W/GaN epitaxial film diodes, η and *ϕ*_*b*0_ are found to be lie between 1.6–1.8 and 0.58–0.61 eV, respectively. For calculating *ϕ*_*b*0_ in the case W/GaN NRs diodes, effective area needs to be re-calculated due to air gap between the individual NRs. Therefore, effective area of tip in contact with NRs array is reduced in comparison to tip contacting GaN epitaxial film. Using density of NRs (=1.86 × 10^9^ *cm*^−2^) and area of top of one nanorod (=3.46 × 10^−10^ *cm*^−2^), effective contact area in NRs is found to 0.64 times of contact area in epitaxial film. The η and *ϕ*_*b*0_ for W/GaN NRs diodes are found to be lying between 4.5–4.8 and 0.50–0.52 eV, respectively. Due to large area contact between W and GaN epitaxial film, electrical field is uniformly distributed beneath the contact which resulted in lesser ideality factor (varying between 1.6–1.8) as compared to ideality factor for nanoscale W/GaN diodes (4.5–4.8 for diode fabricated on array of NRs and 3.3 for diode on single NR). It is well known that electronic transport across MS interface is affected by interface states, (*N*_*SS*_) which are distributed in both space and energy. Under forward bias, a part of applied voltage is dropped across these interface states which results in lesser voltages available for carries undergoing thermionic emission causing η to increase. Therefore, higher η in nanoscale diodes (W/GaN NRs array or W/GaN single NR) in comparison to epitaxial diodes implies higher *N*_*SS*_ in nanoscale diodes which is also expected due to ICP-RIE treatment. As seen in Fig. 5(c), the leakage current is higher for diodes fabricated on NRs array in comparison to diodes on epitaxial film. The reason for leakage current in Schottky diodes is the surface states which act as the recombination centers or favour the trap-assisted tunneling and provide the leakage path to carriers giving rise to leakage current^46^. Due to large surface area in NRs, more surface recombinations take place. Higher the recombinations through these surface states, higher the leakage current in Schottky diodes fabricated on array of NRs in comparison to epitaxial diodes.

To improve the electrical properties of nanoscale Schottky diodes, density of surface states due to dry etching process needs to be reduced. The one possible solution is the wet etching of GaN NRs in 20% KOH solution for about 10 mins at 40 °C after dry etching as suggested by Debnath *et al*.^28^. The wet etching process removed the damages created on side walls of the NRs during ICP-RIE dry etching process. This resulted in reduction of surface states which enhanced the NBE luminescence. Polyakov *et al*.^29^ also observed increased NBE luminescence in KOH treated GaN NRs. Choi *et al*.^50^ observed the enhancement in electroluminescence intensities of InGaN/GaN NRs after Sulphur passivation. They attributed this improvement to the reduced non-radiative recombinations at NRs side walls. Therefore, processes like wet etching of NRs in KOH solution after ICP-RIE treatment or Sulphur passivation are likely to reduce the density of surface states.

It is well known that barrier inhomogeneities which exists at MS interface significantly reduces the barrier height measured by I-V characteristics^47^,^51^,^52^,^53^. The lower values of *ϕ*_*b*0_ for W/GaN NRs array diodes in comparison to W/GaN epitaxial diodes indicate higher level of barrier inhomogeneities in former as compared to latter. Due to barrier inhomogeneities, a diode can be assumed as consisting of patches having low and high barrier height following Gaussian distribution. Current prefers to flow across low SBH causing effective barrier height to decrease^47^,^53^,^54^. Therefore, in the case of W/GaN epitaxial and W/NRs diode, we get lower *ϕ*_*b*0_, but in case of diode fabricated on a single NR, role of barrier inhomogeneities will be less as contact area between W tip and GaN single NR is itself on nanoscale dimensions. Thus, a higher *ϕ*_*b*0_ (0.68 eV) is obtained for Schottky diode on a single GaN nanorod.

#### 1/*f* noise properties of Schottky barrier diodes on GaN NRs array and GaN epitaxial film

**Figure 6:**
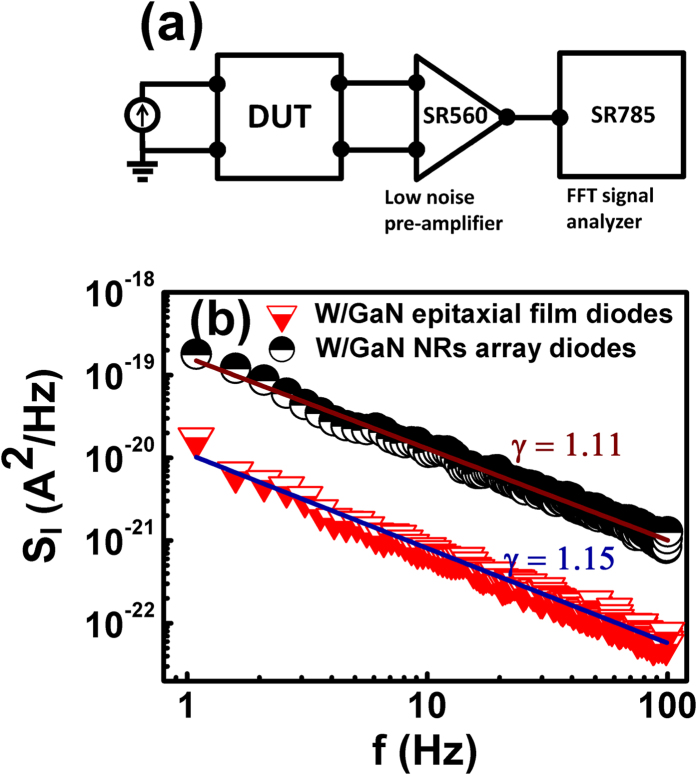
(**a**) Schematic of 1/*f* noise measurement set-up (**b**) Variation of spectral power density of current fluctuations with frequency at room temperature for W/GaN epitaxial film and NRs diodes. The values of γ lying between 1.1–1.2 confirm 1/*f* behaviour of noise spectral density. Here, the spectrum is obtained by averaging noise spectra at four different regions in GaN epilayer as well as NRs.

To further investigate the electronic transport at nanoscale Schottky junctions, 1/*f* noise measurements are carried out on W/GaN epitaxial film diodes as well as W/GaN NRs array diodes. We could not perform the noise measurements in diode fabricated on single nanorod due to complexities of experimental set up i.e. probing a single nanorod inside FESEM which can generate excessive background noise as well as current flowing in the case of single GaN nanorod diode is very low (~nA or pA) which is beyond the detection of our noise set up. It is well established that 1/*f* noise measurements coupled with other electrical characterization techniques can give substantial information about the nature of electrical transport in bulk as well as nanoscale junctions^47^,^55^. The 1/*f* noise measurements are performed at room temperature in the frequency range of 1 to 100 Hz where a battery generated forward current of 1 μA is applied across the epitaxial and NRs array diodes. Schematic of 1/*f* noise experiment for epitaxial film and NRs diodes is shown in Fig. 6(a). In 1/*f* noise measurements, the variation of spectral power density of voltage fluctuations (*S*_*V*_) with frequency is measured. The *S*_*V*_ is then converted into spectral power density of current fluctuations (*S*_*I*_) as^47^,^53^,^54^,^56^.


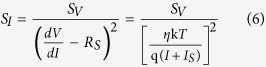


where symbols have usual meaning. For reliable measurements, noise measurements are performed on four diodes realized by probing W tip at four different regions in epitaxial film as well as NRs array diodes. For a single noise spectrum, 10 noise spectra are averaged. Noise measurements are carried out at four random locations and at each location, measurements are performed four times to get better reliability of data. The noise spectra for W/GaN epitaxial and W/GaN NRs array diodes are shown in Fig. 6(b) which are obtained by averaging noise spectra of all four diodes i.e. 160 spectra. The noise spectra at four random positions for both the diodes are shown in Supplementary information. As shown in Fig. 6(b), *S*_*I*_ followed 1/*f*^* γ*^ with *γ* = 1.15 and 1.11 for W/GaN epitaxial and W/GaN NRs array diodes, respectively. The values of *γ* close to unity confirm 1/*f* noise behaviour in both epitaxial as well as NRs array Schottky diodes. The magnitude of 1/*f* noise is about one order higher in W/NRs array diodes as compared to W/GaN epitaxial films diodes.

The 1/*f* noise behaviour in both the diodes and higher level of noise in W/GaN NRs array diodes can be explained using existence of barrier inhomogeneities and interface states. Madenach and Werner^57^ proposed Lorentzian behaviour of noise in the case of electrically homogenous barrier by assuming interface states having same energy or continuously distributed in energy. For an electrically inhomogeneous barrier, barrier heights fluctuations on nano-meter scale affect the noise properties resulting in deviation of Lorentzian noise spectrum towards 1/*f* noise spectrum. More information about deviation of Lorentzian noise spectra towards 1/*f* noise spectra due to barrier inhomogeneities is given in our previous studies on noise behaviour in Ni/GaN diodes^47^,^54^. Therefore, 1/*f* noise behaviour instead of Lorentzian spectra for W/GaN epitaxial as well as W/GaN NRs diodes in present study is due to barrier inhomogeneities at the MS interface. It is discussed earlier that level of barrier inhomogeneities in W/GaN NRs diodes is higher as compared to W/GaN epitaxial film diodes. Higher level of barrier inhomogeneities implies higher numbers of local barriers participating in conduction. The time constant of random capture-emission of carriers at interface depends exponentially on barrier height. Thus higher the barrier inhomogeneities, higher is the range of time constants contributing to conduction resulting in larger noise^57^. Therefore, 1/*f* noise is higher in W/GaN NRs diodes in comparison to epitaxial diodes. Another factor which affects the noise behaviour is the density of interface states, *N*_*SS*_. Under a bias, movement of Fermi level depends upon interface trap levels resulting in charge modulation in interface states which in turn can affect noise properties of the diode. Various authors established a linear relation between density of interface states, *N*_*SS*_ and noise power density^57^,^58^,^59^. As *N*_*SS*_ is expected to be higher in W/GaN NRs diode due to ICP-RIE treatment (discussed earlier), therefore higher level of 1/*f* noise in W/GaN NRs diode as compared to W/GaN epitaxial diode is attributed to the higher level of barrier inhomogeneities as well as higher density of interface states.

## Conclusions

Vertically standing GaN NRs have been fabricated using Ni nanomasking and reactive ion etching. Relatively defect free top of the rod and limited role of barrier inhomogeneities in Schottky diode based on single NR resulted in improved ideality factor and Schottky barrier height as compared to previous reports of Schottky diodes fabricated on individual GaN nanostructures grown using bottom-up approach. The size of nanoscale diode is compared with zero bias depletion width to explain departure from thermionic emission. The lower surface band bending value revealed higher density of surface states in NRs due to ICP-RIE processing. Effective doping concentration across a single GaN nanorod is found to be higher by about two orders of magnitude as compared to GaN epilayer which enhances the tunnelling causing ideality factor to increase for nanoscale diodes in comparison to large area diodes on parent epitaxial films. The higher 1/*f* noise level in large area diodes fabricated on array of NRs in respect to epitaxial film diodes is attributed to higher level of barrier inhomogeneities and density of interface states due to damages induced during RIE. Present study compares electronic transport in nanoscale diodes with large area diodes and may be useful for advancement of GaN in nano-electronics and nano-photonics.

## Methods

### Fabrication of GaN NRs

Unintentionally doped GaN epitaxial films (1 cm × 1 cm × 7.5 μm) over c-plane sapphire (300 μm thick) grown by metal organic chemical vapor deposition (MOCVD) are used for the present work. Carrier concentration in the epitaxial film is found to be 2 × 10^16^ cm^−3^ at room temperature as obtained from Hall Effect measurements (Ecopia HMS-5000). The epitaxial films are cleaned in acetone and iso-propanol followed by dipping in 1:1 solution of HCl:DI water (32% HCl) to remove any native oxide. Finally the samples are rinsed with DI water and then blown with dry nitrogen. For fabricating GaN NRs, a 100 nm thick SiO_2_ film is deposited on the GaN epilayer using thermal evaporation. Rectangular areas on both sides of the sample (1 cm × 2 mm) are protected using a metal shadow mask from oxide deposition for making Ohmic contacts for device characterization. Next, Ni film of thickness 10 nm is deposited using e-beam evaporation. Rapid thermal annealing (RTA) is performed at 850 °C for 1 min in flowing N_2_ to form Ni nanoparticles (NPs). Next, ICP-RIE of SiO_2_ using CF_4_/SF_6_/H_2_ in the ratio of 10.5/21/1 sccm respectively, followed by RIE of GaN in Cl_2_/Ar plasma in the ratio 25/2 sccm are performed to fabricate GaN NRs. The forward power used for SiO_2_ and GaN etching is 200 W and 40 W, respectively. ICP power of 150 W is used only during GaN etch. During ICP-RIE process, Ni NPs particles acted as the mask for fabricating GaN NRs. After reactive ion etching, wet etching of the sample is performed using 5% HF to remove SiO_2_. Morphologies of resulting Ni NPs and GaN NRs are inspected using field emission scanning electron microscopy (FESEM) (Hitachi S4800) respectively.

### Optical characterizations

Micro-Raman and PL measurements are carried out using a Horiba LabRAM HR Evolution Raman spectrometer. PL measurements are performed in the range of 330 nm to 650 nm, using a He-Cd laser excitation at a wavelength of 325 nm. Raman measurements are performed in the range of 200 to 1000 cm^−1^ using Ar ion laser excitation at a wavelength of 514 nm. CL spectra are acquired at various positions of a single GaN NR using GatanMonoCL in Hitachi S4800 SEM.

### Surface potential measurement using Kelvin probe force Microscopy

KPFM on Bruker’s Dimension ICON AFM is used to record images of surface topography and contact potential difference (*V*_*CPD*_) between the conducting tip and sample surface. Kelvin probe force microscopy (KPFM) measurements are performed using the lift-mode technique. In the lift-mode, the surface topography is acquired in tapping mode along a single line profile. Following this, the mechanical excitation of the cantilever is turned off and a second scan is executed along the same line following the topographic profile at a user specific lift-height (LH) from the sample surface, recording local variations in CPD. During the second scan, the tip-sample distance is constant and is equal to *d*_*AFM*_ + LH, where *d*_*AFM*_ represents the tip-sample distance during the topographic scan. During our measurements LH was kept at 50 nm while an AC bias (*V*_*ac*_) of 500 mV is applied between the tip and sample. No DC bias is applied. Silicon tips coated with conductive Pt-Ir and a radius of 20 nm are used to record KPFM measurements. The spring constant and resonant frequency of these tips was 2.8 N/m and 75 kHz, respectively. Due to their relatively lower spring constant, these tips are known for providing large mechanical deflection compared with various other tapping mode probes. In order to obtain a value for the absolute *V*_*CPD*_ of a single NR, square portions are selected from a single NR which is then used to obtain multiple points across the surface of a single NR.

### I-V, KPFM and 1/*f* noise characterizations

Current-voltage (I-V) characteristics of nanoscale and epitaxial diodes are recorded using a Keithley semiconductor characterization system (SCS-4200). For 1/*f* noise measurements of W/GaN epitaxial and W/GaN NRs diodes, a battery generated direct current of 1 μA is applied across each diode. The voltage generated is ac coupled with a low noise pre-amplifier (Stanford Research Systems, model 560). The signal is then fed to a fast Fourier transform dynamic signal analyser (Stanford Research Systems, model 785) which gives the variation of noise spectral density with frequency. To get the background noise, spectrum is taken at *I* = 0 *A*. Next, spectrum at *I* = 1 *μA* is obtained which contains noise from the device as well as background noise. By subtracting the background noise at zero current from noise at finite current, noise arising purely from device is obtained. The 1/*f* noise measurements are performed at room temperature with frequency varying between 1 and 100 Hz. For one spectrum, an average of 10 spectra is taken.

## Additional Information

**How to cite this article**: Kumar, A. *et al*. Barrier inhomogeneities limited current and 1/f noise transport in GaN based nanoscale Schottky barrier diodes. *Sci. Rep.* **6**, 27553; doi: 10.1038/srep27553 (2016).

## Supplementary Material

Supplementary Information
