# Supplementary material for: Barrier inhomogeneities limited current and 1/f noise transport in GaN based nanoscale Schottky barrier diodes
Source: Sci Rep. 2016 Jun 10;6:27553. doi: 10.1038/srep27553 (PMC4901317; doi:10.1038/srep27553)
Supplement: Supplementary Information [file srep27553-s1.pdf]

Supplementary Information

Barrier inhomogeneities limited current and 1/*f* noise transport in GaN based nanoscale Schottky barrier diodes

Ashutosh Kumar,[1,2*] M. Heilmann,[3] Michael Latzel,[3,4] Raman Kapoor,[2] Intu Sharma,[1] M. Göbelt,[3] Silke H. Christiansen,[4,5] Vikram Kumar,[1,2] and Rajendra Singh [1,2*]

[1]Department of Physics, Indian Institute of Technology Delhi, New Delhi-110016, India.

[2]Nanoscale Research Facility, Indian Institute of Technology Delhi, New Delhi-110016, India.

[3]Max Planck Institute for the Science of Light, Günther-Scharowsky-Straße 1/Bau 24, 91058 Erlangen, Germany.

[4]Friedrich-Alexander-Universität Erlangen-Nürnberg (FAU), Institute of Optics, Information and Photonics, Staudtstr. 7/B2, 91058 Erlangen, Germany.

[5]Institute of Nano-Architectures for Energy Conversion, Helmholtz - Zentrum Berlin für Materialien und Energie GmbH, Hahn-Meitner-Platz 1, 14109 Berlin, Germany.

***** Address correspondence to akphy1@gmail.com, rsingh@physics.iitd.ac.in

Raman measurements

To investigate strain relaxation in GaN nanorods (NRs), Raman measurements have been performed in $z(-,-)\overline{z}$ backscattering geometry (z axis is parallel to c-axis) on GaN epitaxial film and GaN NRs. Group theory predicts six Raman modes (one A_1, two B, one E_1 and two E_2) for GaN. Out of these six modes, Raman selection rules allow only E_2 (High) and A_1 (LO) modes

in this backscattering geometry.[1,2] Fig. S1(a) shows Raman spectra of GaN epilayer and GaN NRs, measured in the range of 500 to 800 cm^{-1} where E_2 (High) and A_1(LO) peaks are centered close to 570 cm^{-1} and 735 cm^{-1}, respectively. For comparison, E_2 (High) peak is plotted on expanded scale for epilayer and NRs, as shown in Fig. S1(b) and (c), respectively. In the case of epilayer, a single E_2 peak appears which is fitted using Lorentzian single peak fitting. The peak is centered at 570.4 cm^{-1} with a full width at half maximum (FWHM) of 2.2 cm^{-1} as shown in Fig. S1(b). For GaN NRs, a broader E_2(high) peak appears which is then de-convoluted into two peaks using Lorentzian multi-peak fitting as shown in Fig. S1(c).

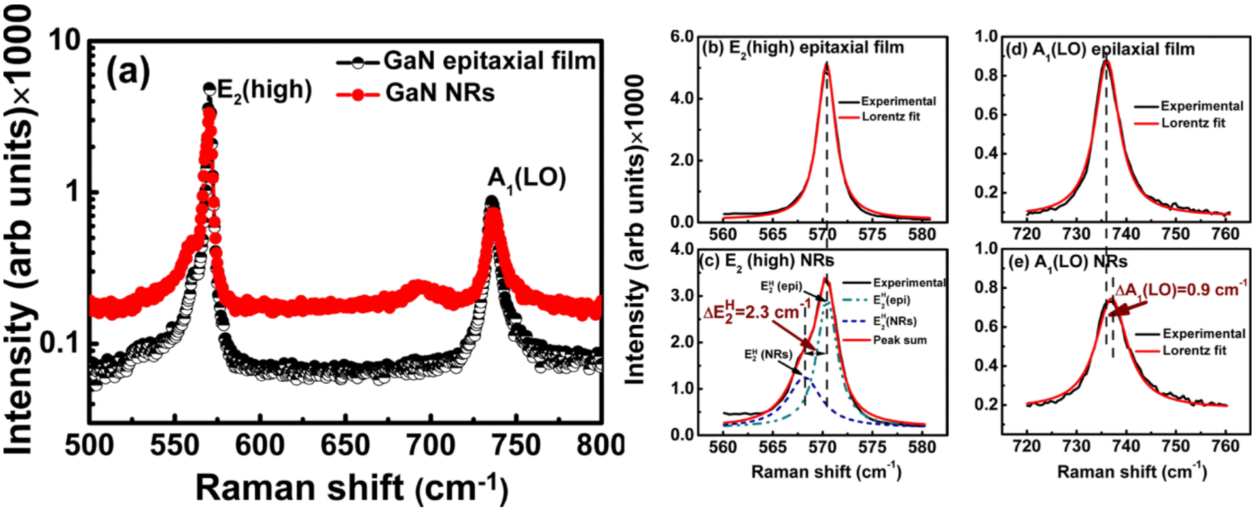

Fig. S1 (a) shows Raman measurement of as-grown GaN epitaxial layer and NRs in the range 500 to 800 cm^{-1}. (b) and (c) show the E_2(high) peak on expanded scale for as-grown epitaxial film and NRs, respectively. E_2(high) peak corresponding to NRs is shifted to lower wave number by 2.3 cm^{-1} as shown in (c) due to stress relaxation. A_1(LO) peaks on expanded scale for both the samples are shown in (d) and (e). Broadening of E_2(high) and A_1(LO) peaks in NRs indicate slight degradation in crystal quality due to ion bombardment during ICP-RIE process.

The peak at 570.5 cm^{-1} labeled as $E_2^H(epi)$ with FWHM of 2.4 cm^{-1} is attributed to Raman signal coming from underlying GaN layer as its position is identical to E_2 peak in epilayer (570.4 cm^{-1}). The other peak labeled as $E_2^H(NRs)$ centered at 568.2 cm^{-1} FWHM of 4.0 cm^{-1} is due to NRs

and is red shifted by 2.3 cm^{-1} as compared to $E_2^H(epi)$. The increased FWHM of $E_2^H(NRs)$ peak in comparison to $E_2^H(epi)$ suggest slight degradation in crystalline quality due to damages created during inductively coupled plasma-reactive ion etching (ICP-RIE).[3] Biaxial residual stress in epilayer as well as NRs can be calculated using frequency shift in E_2(high) peak ΔE_2^H as $\Delta E_2^H\ (cm^{-1}) = \omega - \omega_0 = K\sigma$, where ω and ω_0 are E_2(high) peak positions for measured GaN epilayer (or NRs) and stress free GaN, respectively, K (cm^{-1}/GPa) is proportionality constant and σ is biaxial residual stress.[3] Using the values $K = -2.56\ \mathrm{cm}^{-1}/$ GPa[3,4] and $\omega_0 = 567.6\ \mathrm{cm}^{-1}$ for stress free GaN[3], σ can be calculated for epilayer and NRs in present work. The negative sign in the value of proportionality constant K indicates that compressive nature of stress relaxation. With respect to stress free GaN, values of σ are calculated to be -1.1 GPa for epilayer and -0.2 GPa for NRs. Therefore, a stress relaxation $\big(\Delta\sigma = \sigma(epitaxial\ film) - \sigma(NRs)\big)$ of -0.90 GPa is observed in NRs as compared to epilayer in present work. It is worth mentioning here that different values of K have been used by various authors for calculating the magnitude of stress relaxation due to nano-structuring.[3-5] We have adopted the value of K from the work of Nagarajan *et. al.*[3] where GaN nanorods are fabricated in the similar manner as that of present work. However, we are not using the calculated amount of stress relaxation for any further calculation. This number $\Delta\sigma = -0.90$ GPa only represents that compressive stress is relaxed due to nano-structuring. Furthermore, A_1(LO) peaks for epilayer and NRs are plotted on expanded scale from 720 cm^{-1} to 760 cm^{-1} as shown in Fig. S1(d) and (e), respectively. A_1(LO) peaks for the epilayer and NRs are fitted using Lorentzian single peak fit and found to be centered at 736.1 cm-1 (with FWHM of 6.0 cm^{-1}) and 737.0 cm^{-1} (FWHM of 7.8 cm^{-1}). The FWHM of the A_1(LO) peaks are found to be 6.0 cm^{-1} and 7.8 cm^{-1} for epilayer and NRs, respectively. The ICP-RIE causes ion-induced damages which deteriorates the crystalline

quality resulting in increased FWHM of A_1(LO) peak of NRs sample in comparison to epitaxial layer. The underlying roughened GaN layer causes light to scatter from normal incidence thus shifting polar-sensitive A_1(LO) mode to higher wavenumber side.[3] As level of stress is directly related to the dislocation density, therefore reduced stress in GaN NRs can possibly improve the performance of GaN based nanoscale devices. The strain relaxation also increases the possibility of incorporating more Indium, hence allows the fabrication of more efficient GaN heterostructures based light emitting diodes.[6] The strain relaxation also affects the energy band gap of the semiconductors as discussed in next section i.e. photoluminescence measurements.

Photoluminescence (PL) measurements

Room temperature micro- PL measurements are performed on as-grown GaN epilayer and GaN NRs as shown in Fig. S2(a). The peaks centered close to 3.4 eV for both the samples are re-plotted on expanded scale and de-convoluted into two peaks using Lorentzian multi peak fitting (see Figs S2(b) and (c)). In both the cases, peak 1 shows the near band-edge (NBE) luminescence whereas peak 2 arises due to donor-acceptor pair (DAP) transitions. The peaks corresponding to NBE luminescence (peak 1in Figure S2(b) and (c)) for epitaxial film and NRs are found to be centered at 3.43 and 3.41 eV, respectively. Thus a red shift of about 20 meV in NBE peak from NRs as compared to epilayer is observed which is attributed to compressive stress relaxation in NRs. The stress relaxation ($\Delta\sigma$) is calculated using a proportionality constant, k (=21.2 meV/GPa for stress induced PL peak shift)[7] and found to be 0.94 GPa. The stress relaxation calculated from PL measurements matches that obtained from Raman measurements (0.90 GPa) as discussed earlier. The GaN epitaxial films used in the present work are grown on sapphire and are under compressive strain due to ~ 14 % lattice mismatch between GaN and sapphire. The energy band gap of the semiconductors is affected by the amount and nature of the residual stress

(compressive or tensile). The compressive stress results in increase in band gap whereas tensile stress decreases the band gap. As compressive stress is relaxed in GaN nanorods, therefore peak 1 which corresponds to energy band gap gets shifted to lower value (3.41 eV) as compared to epitaxial film (3.43 eV). The peaks between 3.0-3.35 eV in GaN epitaxial films and nanostructures arise due to DAP transitions and not likely to shift with release of compressive strain as reported by various authors.[8-10]

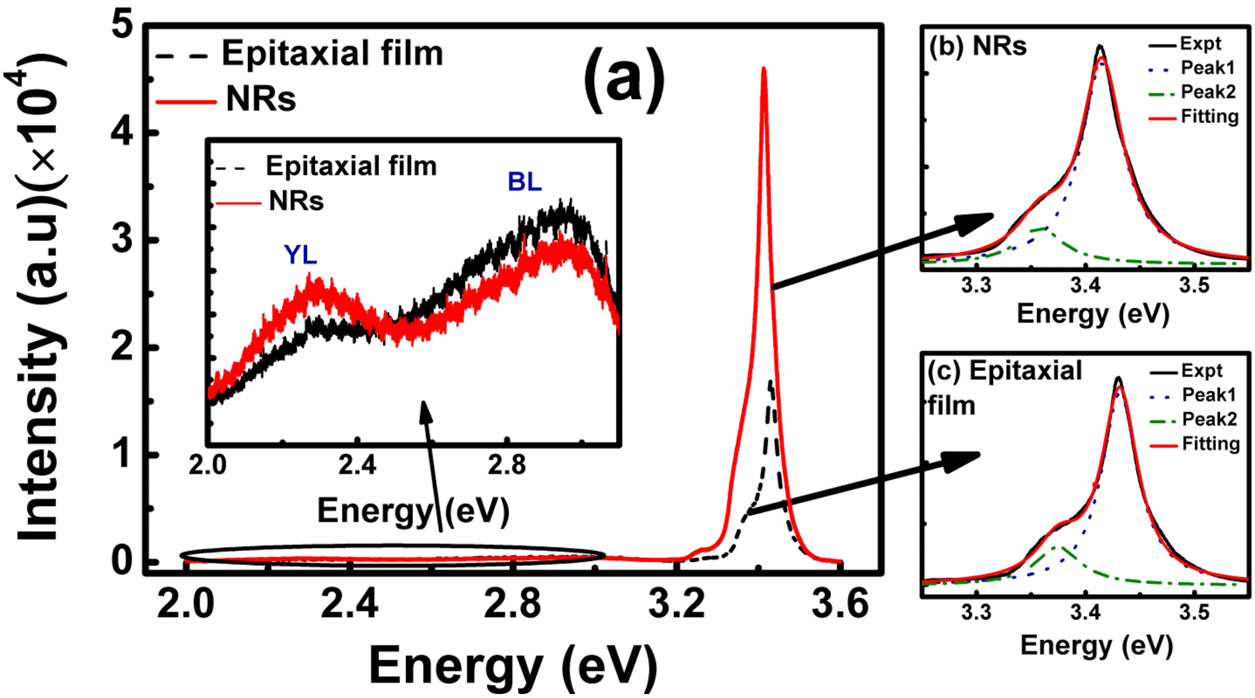

Fig. S2 (a) PL spectra from 2.0 to 3.6 eV of as-grown GaN layer and NRs. Embedded figure in (a) shows the broad YL and BL features around 2.2 and 2.9 eV for GaN epitaxial layer as well as NRs. (b) and (c) show de-convoluted NBE luminescence on expanded scale for NRs and epitaxial film, respectively. A blue shift of about 20 meV in NBE luminescence for NRs as compared to as-grown parent epitaxial film indicates strain relaxation in NRs.

The PL intensity of NBE luminescence is enhanced in the case of NRs as compared to epilayer which may be due to combined contribution from top and side walls of NRs as well as underlying GaN epitaxial film resulting in increased surface area. The enhanced intensity from

NRs could also be due to change in quantum efficiency or different light absorption and collection efficiency. Reddy *et. al.*[11] and Zhuang *et. al.*[12] showed similar results of enhanced NBE luminescence of GaN NRs fabricated using top-down approach in comparison to epilayer. Reddy *et. al.*[11] used finite difference time domain (FTTD) simulations to explain the enhanced NBE luminescence from array of GaN NRs. The reflection losses for array of NRs were found to be lower (~0.25 %) than epitaxial film (19.3 %) which resulted in enhanced light absorption and increased light extraction. Zhuang *et. al.*[12] also employed FTTD simulations to study the distribution of electromagnetic energy along a NR and found that electromagnetic energy is concentrated at the bottom of the NRs. This causes an increase in spontaneous emission rate at such places and thus increases the luminescence. Another possibility of change in NBE luminescence may be change in doping concentration. Schubert *et. al.*[13] reported that intensity of NBE luminescence in GaN epitaxial film increases linearly with doping concentration. In a n-type semiconductor, ratio of radiative to non-radiative recombination rates equals to N_D/N_T, where N_D and N_T are doping and traps concentrations, respectively. If N_D and N_T are independent of each other, increase in N_D increases radiative transitions and thus increases NBE luminescence. Therefore, combined effect of increased surface area, lower reflection losses, increased spontaneous emission rates and increased doping concentration resulted in enhanced NBE in NRs as compared to epitaxial films. Further investigations are required to comment on the relative dominance of these mechanisms. The increase in doping concentration in NRs due to ICP-RIE processing in the present work is verified from KPFM measurements. A broad PL feature from 2.0 to 3.0 eV is also embedded in Fig. S2(a) where two broad peaks centered close to 2.2 eV and 2.9 eV are observed for as-grown epitaxial film as well as NRs. Previous studies reported the peaks lying close to 2.90 eV as blue luminescence (BL).[14] The peak at 2.2 eV,

commonly known as yellow luminescence (YL) arises due to various defects in GaN.[7,15] The findings of the present work are also supported by an earlier work where authors observed NBE peaks at 3.421 eV and 3.410 corresponding to GaN epilayer and NRs, respectively.[7] Enhanced NBE luminescence in NRs with respect to epilayer suggests that these NR arrays may have potential applications in GaN based nanoscale optoelectronic devices.

1/*f* noise measurements for diodes fabricated using tungsten (w) tip at four different positions of GaN epitaxial film and GaN NRs.

For reliable measurements, noise measurements are performed on four diodes fabricated on epitaxial film as well as nanorods. These diodes are realized by contacting W tip at four different positions in GaN epilayer and nanorods. At each region, noise measurements are repeated four times. At each region in epitaxial film as well as NRs, (i.e. for each diode), noise measurements are repeated four times. The spectral power density of current fluctuations as a function of frequency for W/GaN thin films and W/GaN NRs diodes is shown in Fig. S3. Each figure represents an average of four noise spectra.

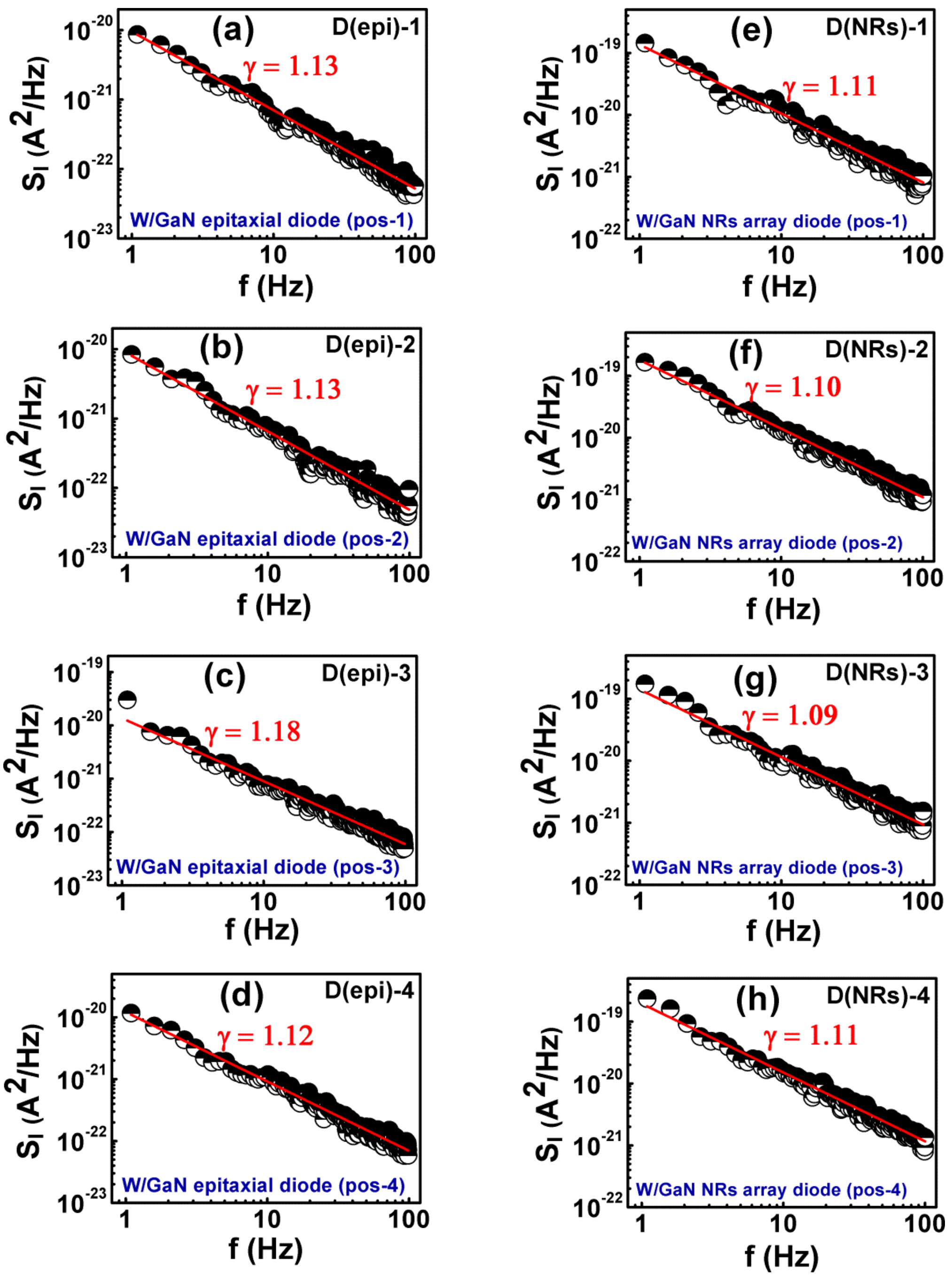

Fig. S3 Variation of spectral power density of current fluctuations with frequency at room temperature for four diodes realized by contacting W tip at four different regions in GaN epilayer (D(epi)-1, D(epi-2), D(epi-3) and D(epi-4) as well as nanorods (D(NRs)-1, D(NRs)-2, D(NRs)-3 and D(NRs)-4). The values of γ lying between 1.1-1.2 confirm 1/*f* behaviour of noise.

References

1 Sarau, G., Heilmann, M., Latzel, M. & Christiansen, S. Disentangling the effects of nanoscale structural variations on the light emission wavelength of single nano-emitters: InGaN/GaN multiquantum well nano-LEDs for a case study. *Nanoscale* **6**, 11953-11962 (2014).

2 Bhattacharya, S., Datta, A., Dhara, S. & Chakravorty, D. Surface optical Raman modes in GaN nanoribbons. *J. Raman Spectrosc* **42**, 429-433 (2011).

3 Nagarajan, S., Svensk, O., Lehtola, L., Lipsanen, H. & Sopanen, M. Stress distribution in GaN nanopillars using confocal Raman mapping technique. *Appl. Phys. Lett.* **104**, 151906 (2014).

4 Wagner, J.-M. & Bechstedt, F. Phonon deformation potentials of α-GaN and -AlN: An ab initio calculation. *Appl. Phys. Lett.* **77**, 346-348 (2000).

5 Duan, H. *et al.* Characterization of GaN grown on 4H-SiC and sapphire by Raman spectroscopy and high resolution XRD. *J. Semicond.* **30**, 073001 (2009).

6 Ra, Y.-H., Navamathavan, R., Park, J.-H. & Lee, C.-R. Coaxial InxGa1–xN/GaN Multiple Quantum Well Nanowire Arrays on Si(111) Substrate for High-Performance Light-Emitting Diodes. *Nano Lett.* **13**, 3506-3516 (2013).

7 Wang, Y. D. *et al.* High optical quality GaN nanopillar arrays. *Appl. Phys. Lett.* **86**, 071917 (2005).

8 Díaz-Guerra, C., Piqueras, J., Castaldini, A., Cavallini, A. & Polenta, L. Defect assessment of Mg-doped GaN by beam injection techniques. *J. Appl. Phys.* **94**, 7470-7475 (2003).

9 Mattila, T. & Nieminen, R. M. Point-defect complexes and broadband luminescence in GaN and AlN. *Phys. Rev. B* **55**, 9571-9576 (1997).

10 Viswanath, A. K. *et al.* Magnesium acceptor levels in GaN studied by photoluminescence. *J. Appl. Phys.* **83**, 2272-2275 (1998).

11 Reddy, N. P. *et al.* Enhanced luminescence from GaN nanopillar arrays fabricated using a top-down process. *Nanotechnology* **27**, 065304 (2016).

12 Zhe, Z. *et al.* Large-scale fabrication and luminescence properties of GaN nanostructures by a soft UV-curing nanoimprint lithography. *Nanotechnology* **24**, 405303 (2013).

13 Schubert, E. F., Goepfert, I. D., Grieshaber, W. & Redwing, J. M. Optical properties of Si-doped GaN. *Appl. Phys. Lett.* **71**, 921-923 (1997).

14 Haab, A. *et al.* Evolution and characteristics of GaN nanowires produced via maskless reactive ion etching. *Nanotechnology* **25**, 255301 (2014).

15 Debnath, R. *et al.* Top-down fabrication of large-area GaN micro- and nanopillars. *J. Vac. Sci. Technol., B* **32**, 021204 (2014).
